# K_3_Al_2_As_3_O_12_

**DOI:** 10.1107/S1600536812000438

**Published:** 2012-01-11

**Authors:** Berthold Stöger, Matthias Weil

**Affiliations:** a Institute for Chemical Technologies and Analytics, Division of Structural Chemistry, Vienna University of Technology, Getreidemarkt 9/164-SC, A-1060 Vienna, Austria

## Abstract

Single crystals of K_3_Al_2_As_3_O_12_, tripotassium dialuminotriarsenate(V), were obtained unintentionally by the reaction of KAsO_3_ with a corundum crucible at 973 K. The asymmetric unit contains three K, two Al, three As and 12 O atoms. The structure of the title compound is isotypic with those of other K_3_ *M*′_2_ *X*_3_O_12_ (*M*′ = Al, Ga; *X* = P, As) structures and is made up of a three-dimensional network of corner-sharing [AlO_4_] and [AsO_4_] tetra­hedra. The three K^+^ cations are located in channels running along the [100], [001], [101] and [10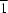] directions, exhibiting different coordination numbers of 9, 8 and 6, respectively. All corresponding [KO_*x*_] polyhedra are considerably distorted.

## Related literature

For a recent review on NASICON-type materials, see: Anantharamulu *et al.* (2011 ▶). For isotypic K_3_ *M*′_2_ *X*_3_O_12_ structures, see: Beaurain *et al.* (2008 ▶) and Yakubovich *et al.* (2008 ▶) for K_3_Ga_2_P_3_O_12_; Boughzala *et al.* (1997 ▶) for the solid solution K_3_Al_2_(As_1.92_P_1.08_)O_12_; Devi & Vidyasagar (2000 ▶) for K_3_Al_2_P_3_O_12_. For the isopointal structure of Tl_3_Al_2_P_3_O_12_, see: Devi & Vidyasagar (2000 ▶). For background to the bond-valence method, see: Brown & Altermatt (1985 ▶).

## Experimental

### 

#### Crystal data

K_3_Al_2_As_3_O_12_
*M* *_r_* = 588
Orthorhombic, 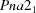

*a* = 8.7943 (2) Å
*b* = 17.4400 (2) Å
*c* = 8.6610 (3) Å
*V* = 1328.36 (6) Å^3^
*Z* = 4
Mo *K*α radiation
μ = 8.63 mm^−1^
*T* = 100 K
0.10 × 0.06 × 0.01 mm

#### Data collection

Bruker APEXII CCD diffractometer
Absorption correction: multi-scan (*SADABS*; Bruker, 2008 ▶) *T*_min_ = 0.49, *T*_max_ = 0.92
52293 measured reflections
9623 independent reflections
8606 reflections with *I* > 3σ(*I*)
*R*_int_ = 0.038

#### Refinement

*R*[*F* ^2^ > 3σ(*F* ^2^)] = 0.018
*wR*(*F* ^2^) = 0.039
*S* = 0.80
9623 reflections
181 parameters
Δρ_max_ = 0.33 e Å^−3^
Δρ_min_ = −0.31 e Å^−3^
Absolute structure: Flack (1983 ▶), 3882 Friedel pairs
Flack parameter: 0.008 (3)

### 

Data collection: *APEX2* (Bruker, 2008 ▶); cell refinement: *SAINT-Plus* (Bruker, 2008 ▶); data reduction: *SAINT-Plus*; method used to solve structure: coordinates taken from an isotypic structure; program(s) used to refine structure: *JANA2006* (Petříček *et al.*, 2006 ▶); molecular graphics: *ATOMS* (Dowty, 2006 ▶); software used to prepare material for publication: *publCIF* (Westrip, 2010 ▶).

## Supplementary Material

Crystal structure: contains datablock(s) global, I. DOI: 10.1107/S1600536812000438/gw2112sup1.cif

Structure factors: contains datablock(s) I. DOI: 10.1107/S1600536812000438/gw2112Isup2.hkl

Additional supplementary materials:  crystallographic information; 3D view; checkCIF report

## Figures and Tables

**Fig. 1.**
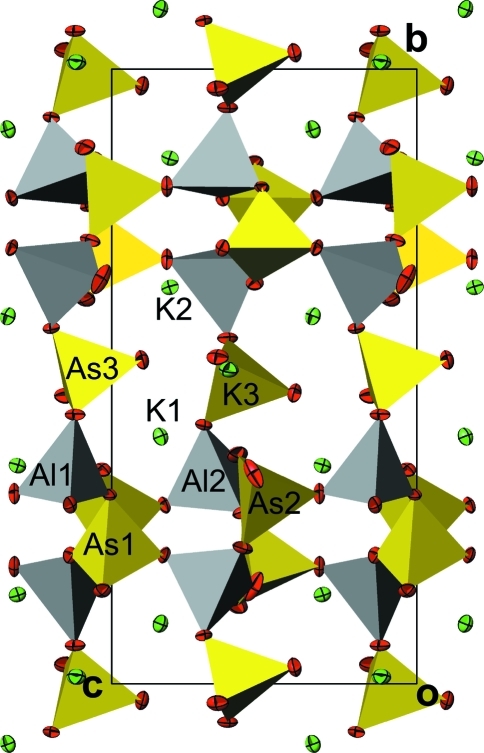
The crystal structure of K3Al2As3O12 viewed down [100], showing sheets of corner-sharing [AsO4] and [AlO4] tetrahedra extending parallel to (010). Displacement ellipsoids are drawn at the 90% probability level.

**Fig. 2.**
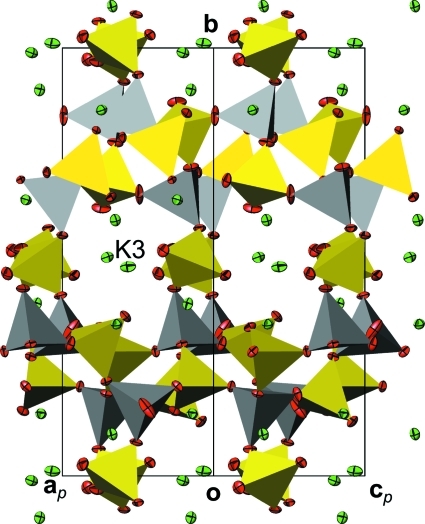
The crystal structure of K3Al2As3O12 viewed down [101]. Displacement ellipsoids are drawn at the 90% probability level.

## supplementary crystallographic information

### Comment

During crystal growth studies of KAsO_3_, we inadvertently obtained single crystals with composition K_3_Al_2_As_3_O_12_ from an attacked corundum crucible. Many oxides with general formula *M_x_M*'_2_*X*_3_O_12_ crystallize with three-dimensional framework structures (Devi & Vidyasagar, 2000) and are of technological interest. Most notably, compounds crystallizing in the NASICON (Na_3_Zr_2_Si_2_PO_12_) structure type are excellent ion conductors and have been intensely studied. A recent review on compounds with the NASICON structure has been given by Anantharamulu *et al.* (2011).

The structure of K_3_Al_2_As_3_O_12_ is isotypic with the phosphate analogue K_3_Al_2_P_3_O_12_ (Devi & Vidyasagar, 2000), the mixed arsenate/phosphate solid solution K_3_Al_2_As_1.92_P_1.08_O_12_ (Boughzala *et al.*, 1997) and K_3_Ga_2_P_3_O_12_ (Beaurain *et al.*, 2008; Yakubovich *et al.*, 2008). Trithallium dialuminotriphosphate, Tl_3_Al_2_P_3_O_12_ (Devi & Vidyasagar, 2000), can be considered as isopointal to the title compound, because it features distinctly different coordinations of the Al sites and the cationic network due to the electron lone pairs of the Tl^+^ ions.

Whereas in the NASICON structure type the *M*' site is octahedrally and the *X* site tetrahedrally coordinated, in the title compound both sites exhibit a tetrahedral coordination. Two crystallographically different [AlO_4_] and three [AlO_4_] tetrahedra, all on general positions, are linked *via* their corners to a complex three-dimensional network, whereby [AlO_4_] units connect only to [AsO_4_] units and *vice*-*versa*. This network can be decomposed into undulating sheets normal to [010] (Al1, Al2, As1, As2) which are connected by [AsO_4_] units (As3) (Fig. 1).

The three different K^+^ cations are located in channels running along the [100] and [001] (K1, K2) (Fig. 1) as well as the [101] and [101] (K3) (Fig. 2) directions. Considering K–O distances up to 3.5 Å as relevant for first coordination spheres, the K^+^ cations are coordinated by 9 (K1), 8 (K1) and 6 (K3) O atoms, respectively, all in the form of irregular [KO*_x_*] polyhedra. The total bond valence sums (parameters: *R*_0_ = 2.132 Å, *b* = 0.37 (Brown & Altermatt, 1985)), 1.08 (K1), 1.04 (K2) and 0.90 (K3) valence units (v.u.) are close to the expected value of 1 v.u. and point to a slight undersaturation of K3. The coordination of the K^+^ cations is very similar in all isotypic structures. The main difference in these structures pertains to the bond lengths of the *X*O_4_ tetrahedra. Corresponding mean bond lengths are 1.746 Å for AlO_4_ and 1.680 Å for AsO_4_ tetrahedra in the title compound; 1.737 Å for AlO_4_ and 1.527 Å for PO_4_ tetrahedra in K_3_Al_2_P_3_O_12_; 1.730 Å for AlO_4_ and 1.615 Å for (As/P)O_4_ tetrahedra in K_3_Al_2_As_1.92_P_1.08_O_12_; 1.816 Å for GaO_4_ and 1.535 Å for PO_4_ tetrahedra in K_3_Ga_2_P_3_O_12_.

### Experimental

K_2_CO_3_ and H_3_AsO_4_ were obtained commercially and used without purification. 10 g 80%_wt_ H_3_AsO_4_ were titrated against an aqueous K_2_CO_3_ solution using methyl red as indicator. The water was evaporated and the residue recrystallized from water to obtain KH_2_AsO_4_. This solid was then heated in a corundum crucible at 973 K, cooled to 633 K over 24 h and quenched. Few colourless crystals of the title compound were isolated from the reaction mixture.

### Refinement

The first refinement cycle was performed using the published atomic coordinates of K_3_Al_2_As_1.92_P_1.08_O_12_ (Boughzala *et al.*, 1997) as starting parameters.

### Crystal data

**Table:**

K_3_Al_2_As_3_O_12_	*F*(000) = 1112
*M**_r_* = 588	*D*_x_ = 2.939 Mg m^−^^3^
Orthorhombic, *P**n**a*2_1_	Mo *K*α radiation, λ = 0.71073 Å
Hall symbol: P 2c -2n	Cell parameters from 54411 reflections
*a* = 8.7943 (2) Å	θ = 3.3–44.8°
*b* = 17.4400 (2) Å	µ = 8.63 mm^−^^1^
*c* = 8.6610 (3) Å	*T* = 100 K
*V* = 1328.36 (6) Å^3^	Plate, colourless
*Z* = 4	0.10 × 0.06 × 0.01 mm

### Data collection

**Table:**

Bruker APEXII CCD diffractometer	9623 independent reflections
Radiation source: X-ray tube	8606 reflections with *I* > 3σ(*I*)
graphite	*R*_int_ = 0.038
ω and φ scans	θ_max_ = 45.1°, θ_min_ = 2.6°
Absorption correction: multi-scan (*SADABS*; Bruker, 2008)	*h* = −17→17
*T*_min_ = 0.49, *T*_max_ = 0.92	*k* = −34→34
52293 measured reflections	*l* = −15→17

### Refinement

**Table:**

Refinement on *F*^2^	Primary atom site location: isomorphous structure methods
*R*[*F*^2^ > 2σ(*F*^2^)] = 0.018	Weighting scheme based on measured s.u.'s *w* = 1/(σ^2^(*I*) + 0.0004*I*^2^)
*wR*(*F*^2^) = 0.039	(Δ/σ)_max_ = 0.003
*S* = 0.80	Δρ_max_ = 0.33 e Å^−^^3^
9623 reflections	Δρ_min_ = −0.31 e Å^−^^3^
181 parameters	Absolute structure: Flack (1983), 3882 Friedel pairs
0 restraints	Flack parameter: 0.008 (3)
1 constraint	

### Fractional atomic coordinates and isotropic or equivalent isotropic displacement parameters (Å^2^)

**Table:**

	*x*	*y*	*z*	*U*_iso_*/*U*_eq_	
As1	0.156896 (11)	0.215743 (6)	0.0009	0.004155 (18)	
As2	0.293241 (12)	0.313601 (6)	0.511929 (16)	0.004909 (19)	
As3	0.236068 (12)	0.502941 (6)	0.085202 (18)	0.005355 (19)	
K1	0.01017 (3)	0.402188 (14)	0.84037 (3)	0.00837 (4)	
K2	0.95033 (3)	0.354962 (14)	0.30906 (3)	0.00818 (4)	
K3	0.68161 (3)	0.488891 (15)	0.11580 (3)	0.01179 (5)	
Al1	0.34851 (4)	0.34012 (2)	0.14668 (4)	0.00492 (7)	
Al2	0.13204 (4)	0.16767 (2)	0.65562 (4)	0.00520 (7)	
O1	0.29089 (10)	0.15184 (5)	0.03110 (9)	0.00824 (17)	
O2	0.02014 (10)	0.20900 (5)	0.13689 (9)	0.00746 (16)	
O3	0.21755 (9)	0.30785 (4)	0.00781 (10)	0.00643 (14)	
O4	0.06929 (10)	0.20939 (5)	0.82779 (9)	0.00730 (16)	
O5	0.17013 (13)	0.37287 (6)	0.58794 (12)	0.0139 (2)	
O6	0.47459 (12)	0.34183 (7)	0.53518 (11)	0.0168 (2)	
O7	0.27956 (11)	0.22138 (5)	0.57055 (11)	0.01044 (18)	
O8	0.26654 (10)	0.30933 (6)	0.31927 (9)	0.00936 (18)	
O9	0.07409 (11)	0.46866 (6)	0.15273 (11)	0.01076 (18)	
O10	0.29028 (11)	0.57855 (5)	0.19818 (10)	0.00894 (17)	
O11	0.23407 (11)	0.52699 (6)	0.90078 (10)	0.01074 (19)	
O12	0.37999 (10)	0.43772 (5)	0.12022 (10)	0.00883 (16)	

### Atomic displacement parameters (Å^2^)

**Table:**

	*U*^11^	*U*^22^	*U*^33^	*U*^12^	*U*^13^	*U*^23^
As1	0.00437 (3)	0.00356 (3)	0.00453 (3)	0.00007 (3)	−0.00035 (3)	−0.00027 (3)
As2	0.00554 (3)	0.00472 (4)	0.00447 (3)	−0.00042 (3)	0.00028 (3)	0.00036 (3)
As3	0.00642 (4)	0.00336 (3)	0.00628 (3)	−0.00018 (3)	−0.00018 (3)	−0.00019 (3)
K1	0.00698 (7)	0.01015 (8)	0.00797 (6)	−0.00039 (6)	0.00040 (5)	0.00102 (6)
K2	0.00765 (8)	0.00734 (8)	0.00955 (7)	0.00014 (6)	0.00017 (6)	0.00078 (6)
K3	0.01516 (10)	0.00792 (9)	0.01230 (8)	−0.00147 (7)	−0.00295 (7)	−0.00020 (6)
Al1	0.00487 (12)	0.00439 (12)	0.00550 (10)	−0.00023 (9)	−0.00019 (9)	−0.00024 (9)
Al2	0.00592 (12)	0.00438 (12)	0.00532 (10)	0.00071 (10)	−0.00068 (9)	−0.00031 (9)
O1	0.0078 (3)	0.0069 (3)	0.0101 (3)	0.0029 (2)	−0.0011 (2)	−0.0004 (2)
O2	0.0062 (3)	0.0094 (3)	0.0067 (2)	−0.0020 (2)	0.0017 (2)	−0.0006 (2)
O3	0.0075 (2)	0.0046 (2)	0.0072 (2)	−0.0014 (2)	−0.0019 (2)	0.0005 (2)
O4	0.0078 (3)	0.0089 (3)	0.0052 (2)	0.0016 (2)	−0.0019 (2)	−0.0019 (2)
O5	0.0193 (4)	0.0088 (3)	0.0138 (3)	0.0037 (3)	0.0083 (3)	−0.0016 (3)
O6	0.0101 (3)	0.0274 (5)	0.0130 (3)	−0.0102 (3)	−0.0069 (3)	0.0108 (3)
O7	0.0121 (3)	0.0056 (3)	0.0136 (3)	0.0006 (2)	0.0041 (2)	0.0026 (2)
O8	0.0094 (3)	0.0144 (4)	0.0043 (2)	−0.0031 (3)	−0.0001 (2)	0.0002 (2)
O9	0.0084 (3)	0.0083 (3)	0.0155 (3)	−0.0023 (2)	0.0031 (2)	0.0001 (3)
O10	0.0131 (3)	0.0043 (3)	0.0094 (3)	−0.0018 (2)	0.0003 (2)	−0.0010 (2)
O11	0.0138 (3)	0.0121 (4)	0.0063 (3)	0.0010 (3)	−0.0004 (2)	0.0016 (2)
O12	0.0085 (3)	0.0044 (3)	0.0136 (3)	0.0006 (2)	−0.0019 (2)	0.0002 (2)

### Geometric parameters (Å, °)

**Table:**

K1—O1^i^	2.7084 (9)	Al1—O8	1.7443 (9)
K1—O3^ii^	2.8524 (8)	Al1—O12	1.7396 (9)
K1—O5	2.6496 (11)	Al2—O4	1.7485 (9)
K1—O9^ii^	2.9964 (10)	Al2—O6^vii^	1.7415 (11)
K1—O9^iii^	2.8747 (10)	Al2—O7	1.7616 (10)
K1—O10^iii^	2.9344 (10)	Al2—O10^viii^	1.7373 (10)
K1—O11	2.9814 (10)	As1—O1	1.6429 (9)
K2—O1^iv^	2.7885 (9)	As1—O2	1.6875 (8)
K2—O2^v^	3.0134 (9)	As1—O3	1.6936 (8)
K2—O7^iv^	3.0260 (10)	As1—O4^ix^	1.6893 (8)
K2—O8^v^	2.8939 (10)	As2—O5	1.6352 (10)
K2—O9^v^	2.6362 (10)	As2—O6	1.6812 (11)
K2—O11^vi^	2.7384 (10)	As2—O7	1.6909 (9)
K3—O1^iv^	2.7360 (9)	As2—O8	1.6867 (8)
K3—O5^vi^	2.7515 (10)	As3—O9	1.6519 (9)
K3—O11^vi^	2.5921 (9)	As3—O10	1.7098 (9)
K3—O12	2.7989 (10)	As3—O11^ix^	1.6515 (9)
Al1—O2^iv^	1.7376 (9)	As3—O12	1.7285 (9)
Al1—O3	1.7578 (9)		
			
O1^i^—K1—O3^ii^	86.82 (3)	O1^iv^—K3—O5^vi^	126.59 (3)
O1^i^—K1—O5	144.51 (3)	O1^iv^—K3—O11^vi^	93.38 (3)
O1^i^—K1—O9^ii^	73.59 (3)	O1^iv^—K3—O12	92.90 (3)
O1^i^—K1—O9^iii^	115.72 (3)	O5^vi^—K3—O11^vi^	92.38 (3)
O1^i^—K1—O10^iii^	69.80 (3)	O5^vi^—K3—O12	136.86 (3)
O1^i^—K1—O11	128.11 (3)	O11^vi^—K3—O12	102.95 (3)
O3^ii^—K1—O5	88.21 (3)	O2^iv^—Al1—O3	112.21 (4)
O3^ii^—K1—O9^ii^	69.16 (3)	O2^iv^—Al1—O8	104.42 (4)
O3^ii^—K1—O9^iii^	154.71 (3)	O2^iv^—Al1—O12	109.74 (5)
O3^ii^—K1—O10^iii^	148.77 (2)	O3—Al1—O8	102.53 (4)
O3^ii^—K1—O11	84.81 (2)	O3—Al1—O12	109.11 (4)
O5—K1—O9^ii^	136.06 (3)	O8—Al1—O12	118.68 (5)
O5—K1—O9^iii^	79.72 (3)	O4—Al2—O6^vii^	107.43 (5)
O5—K1—O10^iii^	98.85 (3)	O4—Al2—O7	111.59 (4)
O5—K1—O11	86.28 (3)	O4—Al2—O10^viii^	108.39 (4)
O9^ii^—K1—O9^iii^	104.81 (3)	O6^vii^—Al2—O7	112.68 (5)
O9^ii^—K1—O10^iii^	120.24 (3)	O6^vii^—Al2—O10^viii^	110.77 (6)
O9^ii^—K1—O11	55.61 (2)	O7—Al2—O10^viii^	105.95 (5)
O9^iii^—K1—O10^iii^	56.01 (3)	O1—As1—O2	110.66 (4)
O9^iii^—K1—O11	72.39 (3)	O1—As1—O3	114.32 (4)
O10^iii^—K1—O11	125.79 (3)	O1—As1—O4^ix^	115.08 (4)
O1^iv^—K2—O2^v^	68.86 (2)	O2—As1—O3	105.42 (4)
O1^iv^—K2—O7^iv^	112.20 (3)	O2—As1—O4^ix^	106.85 (4)
O1^iv^—K2—O8^v^	119.86 (2)	O3—As1—O4^ix^	103.72 (4)
O1^iv^—K2—O9^v^	78.24 (3)	O5—As2—O6	113.25 (6)
O1^iv^—K2—O11^vi^	89.12 (3)	O5—As2—O7	115.67 (5)
O2^v^—K2—O7^iv^	95.73 (2)	O5—As2—O8	109.52 (5)
O2^v^—K2—O8^v^	65.61 (2)	O6—As2—O7	108.07 (5)
O2^v^—K2—O9^v^	107.28 (3)	O6—As2—O8	105.28 (4)
O2^v^—K2—O11^vi^	154.06 (3)	O7—As2—O8	104.19 (5)
O7^iv^—K2—O8^v^	109.45 (3)	O9—As3—O10	108.48 (5)
O7^iv^—K2—O9^v^	156.96 (3)	O9—As3—O11^ix^	115.16 (5)
O7^iv^—K2—O11^vi^	79.62 (3)	O9—As3—O12	109.34 (4)
O8^v^—K2—O9^v^	79.97 (3)	O10—As3—O11^ix^	111.13 (5)
O8^v^—K2—O11^vi^	140.08 (3)	O10—As3—O12	101.71 (4)
O9^v^—K2—O11^vi^	80.10 (3)	O11^ix^—As3—O12	110.16 (5)

Symmetry codes: (i) *x*−1/2, −*y*+1/2, *z*+1; (ii) *x*, *y*, *z*+1; (iii) −*x*, −*y*+1, *z*+1/2; (iv) *x*+1/2, −*y*+1/2, *z*; (v) *x*+1, *y*, *z*; (vi) −*x*+1, −*y*+1, *z*−1/2; (vii) *x*−1/2, −*y*+1/2, *z*; (viii) −*x*+1/2, *y*−1/2, *z*+1/2; (ix) *x*, *y*, *z*−1.
